# An experimental study of animating-based facial image manipulation in online class environments

**DOI:** 10.1038/s41598-023-31408-y

**Published:** 2023-03-22

**Authors:** Jeong-Ha Park, Chae-Yun Lim, Hyuk-Yoon Kwon

**Affiliations:** 1 Graduate School of Data Science, Seoul National University of Science and Technology, Seoul, South Korea; 2 Department of Industrial Engineering, Seoul National University of Science and Technology, Seoul, South Korea; 3 Department of Industrial Engineering/ Graduate School of Data Science/ Research Center for Electrical and Information Science, Seoul National University of Science and Technology, Seoul, South Korea

**Keywords:** Computer science, Information technology

## Abstract

Recent advances in artificial intelligence technology have significantly improved facial image manipulation, which is known as Deepfake. Facial image manipulation synthesizes or replaces a region of the face in an image with that of another face. The techniques for facial image manipulation are classified into four categories: (1) entire face synthesis, (2) identity swap, (3) attribute manipulation, and (4) expression swap. Out of them, we focus on expression swap because it effectively manipulates only the expression of the face in the images or videos without creating or replacing the entire face, having advantages for the real-time application. In this study, we propose an evaluation framework of the expression swap models targeting the real-time online class environments. For this, we define three kinds of scenarios according to the portion of the face in the entire image considering actual online class situations: (1) attendance check (Scenario 1), (2) presentation (Scenario 2), and (3) examination (Scenario 3). Considering the manipulation on the online class environments, the framework receives a single source image and a target video and generates the video that manipulates a face of the target video to that in the source image. To this end, we select two models that satisfy the conditions required by the framework: (1) first order model and (2) GANimation. We implement these models in the framework and evaluate their performance for the defined scenarios. Through the quantitative and qualitative evaluation, we observe distinguishing properties of the used two models. Specifically, both models show acceptable results in Scenario 1, where the face occupies a large portion of the image. However, their performances are significantly degraded in Scenarios 2 and 3, where the face occupies less portion of the image; the first order model causes relatively less loss of image quality than GANimation in the result of the quantitative evaluation. In contrast, GANimation has the advantages of representing facial expression changes compared to the first order model. Finally, we devise an architecture for applying the expression swap model to the online video conferencing application in real-time. In particular, by applying the expression swap model to widely used online meeting platforms such as Zoom, Google Meet, and Microsoft Teams, we demonstrate its feasibility for real-time online classes.

## Introduction

Due to the recent development of artificial intelligence technology, the performance of facial image manipulation technology, widely known as Deepfake, has greatly improved^1–3^. Facial image manipulation is a type of digital image manipulation^4^ and refers to a technique of synthesizing or replacing a face within an image with another face. Various techniques for manipulating facial images, such as morphing^5,6^, swapping^7,8^, and retouching^9,10^, have been proposed. Fake images or videos generated by facial image manipulation are rapidly spread and can be utilized in news or social network services. This can be used positively in various fields such as movies and albums due to its high commercial value, but if it is abused for crimes such as pornography and fake news, it can cause serious social problems. Due to the outbreak of COVID-19, online lectures using video conferencing programs such as Zoom and Google Meet have become popular, and they also could be a major target for facial image manipulation techniques.

Cheating in an online class is difficult to fully detect, and furthermore, finding concrete evidence of cheating is very challenging^11^. As a result, it has been usually handled through human-led online proctoring. Previous studies on cheating detection in online exams have relied on statistical evidence, such as the time of answering questions, browser changes, and typing speed^12^. An e-exam management system using a fingerprint reader and eye tracker has also been proposed^13^. With the shift to online education due to the COVID-19 pandemic, several studies on online cheating detection have emerged such as Py-Cheat(https://github.com/abalderas/Py-Cheat), a Python tool that detects groups of students who may have cheated together based on variables such as start and completion time, grades, and classes. However, these methods cannot address cheating through facial image manipulation because it cannot be detected through the student’s actions.

In this study, we evaluate the performance of facial image manipulation techniques targeting online class environments, which have not been considered before. Table 1 shows a taxonomy of the existing models for facial image manipulation. Techniques for facial image manipulation are largely divided into four categories^14^: (1) entire face synthesis, (2) identity swap, (3) attribute manipulation, and (4) expression swap. In the table, we specified the main approaches for each category and the representative studies. Entire face synthesis creates a face that does not actually exist. GAN-based models^15–18^ have been widely adopted. Identity swap converts a specific person’s face into another person’s one. Traditionally, the computer graphics-based approach^19^ was representative, but recently, with the development of deep neural networks, many deep learning-based models^20–24^ have been proposed. Attribute manipulation changes target attributes such as gender, age, skin color, and whether glasses are worn, rather than changing the face itself. GAN-based generative models^25,26–29^ have been presented to generate new features. Expression swap aims to change facial expressions or movements within an image or video.

**Table 1:** Taxonomy of facial image manipulation.

	Facial image manipulation					
Categories	Entire face synthesis	Identity swap		Attribute manipulation	Expression swap (face reenactment)	
Approaches	GAN-based	Computer graphic-based	Deep learning based	GAN-based	Learning-based	Animating-based
Existing Rese arches	^15–18^	^19^	^20–24^	^25,25–29^	^30–33^	^34–36^

In this study, we focus on expression swap, which is a type of facial image manipulation that allows for changing only the facial expression within an image. Expression swap has the potential for misuse in online class environments because it allows for real-time and realistic changes to facial features^37^. Furthermore, combining expression swap with another technique, such as whole-body skeletal motion, can enhance the quality of anomalous behavior generation^38^. Specifically, a recent study combined identity swap and expression swap into a unified framework, resulting in high-fidelity results^39^. Because the other techniques can be treated as orthogonal issues, we concentrate on expression swap in this study.

We divide expression swap into the following two approaches: 1) learning-based swap^30–33^ and 2) animating-based swap^34–36^. The learning-based swap exhibits excellent performance only for the learned objects because it learns on a sequence of frames for a specific object. On the other hand, the animating-based swap reproduces the expression in the target video according to the face in the source image. It can be accomplished by extracting facial expression movements from target videos and animating facial expressions of source images without learning the objects in the target video. As a result, it has the following advantages to be applied to real-time online environments: 1) it requires only one source image compared to the learning-based approaches, which require sufficient numbers of source images for learning, and 2) it can be generally applied to any input source image. Therefore, we select the animating-based expression swap for the target approach. To this end, we select two animating-based expression swap models that satisfy the conditions required by the framework: 1) first order model^34,35^ and 2) GANimation^36^.

In this study, we aim to answer the following research questions regarding the two representative expression swap models.

**RQ1**. What is the level of realism of the results produced by the expression swap models in actual online environments?

**RQ2**. How does the performance of the expression swap models vary under the different scenarios where the proportion of the face in the whole image changes?

**RQ3**. Is the expression swap model feasible for use in online class environments, taking into account its processing latency?

In order to answer these questions, we propose an evaluation framework for the expression swap models targeting real-time online class environments. For this, we define three kinds of scenarios according to the portion of the face in the entire image considering the actual online class situations: (1) attendance check (Scenario 1), (2) presentation (Scenario 2), and (3) examination (Scenario 3). Considering the manipulation in the online class environments, the framework receives a single source image and a target video and generates the video that manipulates a target video to replace a face in the target video with a face in the source image. We implement these models in the framework and evaluate their performance for the defined scenarios. Through the quantitative and qualitative evaluation, we observe the distinguishing properties of the two models. Specifically, both models show acceptable results in Scenario 1, where the face occupies a large portion of the image. However, their performances are significantly degraded in Scenarios 2 and 3, where the face occupies less portion of the image; the first order model causes relatively less loss of image quality than GANimation in the result of the quantitative evaluation. In contrast, GANimation has the advantage of representing the facial expression changes compared to the first order model. Finally, we devise an architecture for applying the expression swap model to the online video conferencing application in real-time. Using the Zoom application, we observed that the processing delay was only 0.3 s for first order model and 0.5 s for GANimation model, demonstrating its effectiveness for real-time applications.

The paper is organized as follows. In “Related work” section, we present related work. In “Evaluation framework for facial image manipulation” section, we explain the animating-based expression swap models applied for facial image manipulation. In “Scenarios for online class environments”, we define three scenarios that can occur in online class situations. In “‘Performance evaluation” section, we show the performance evaluation results in qualitative and quantitative ways. In “Demonstration” section, we propose the actual demonstration method to show the real-time usability of our framework. In “Conclusions” section, we conclude the paper and discuss future research directions.

## Related work

**Figure 1:**
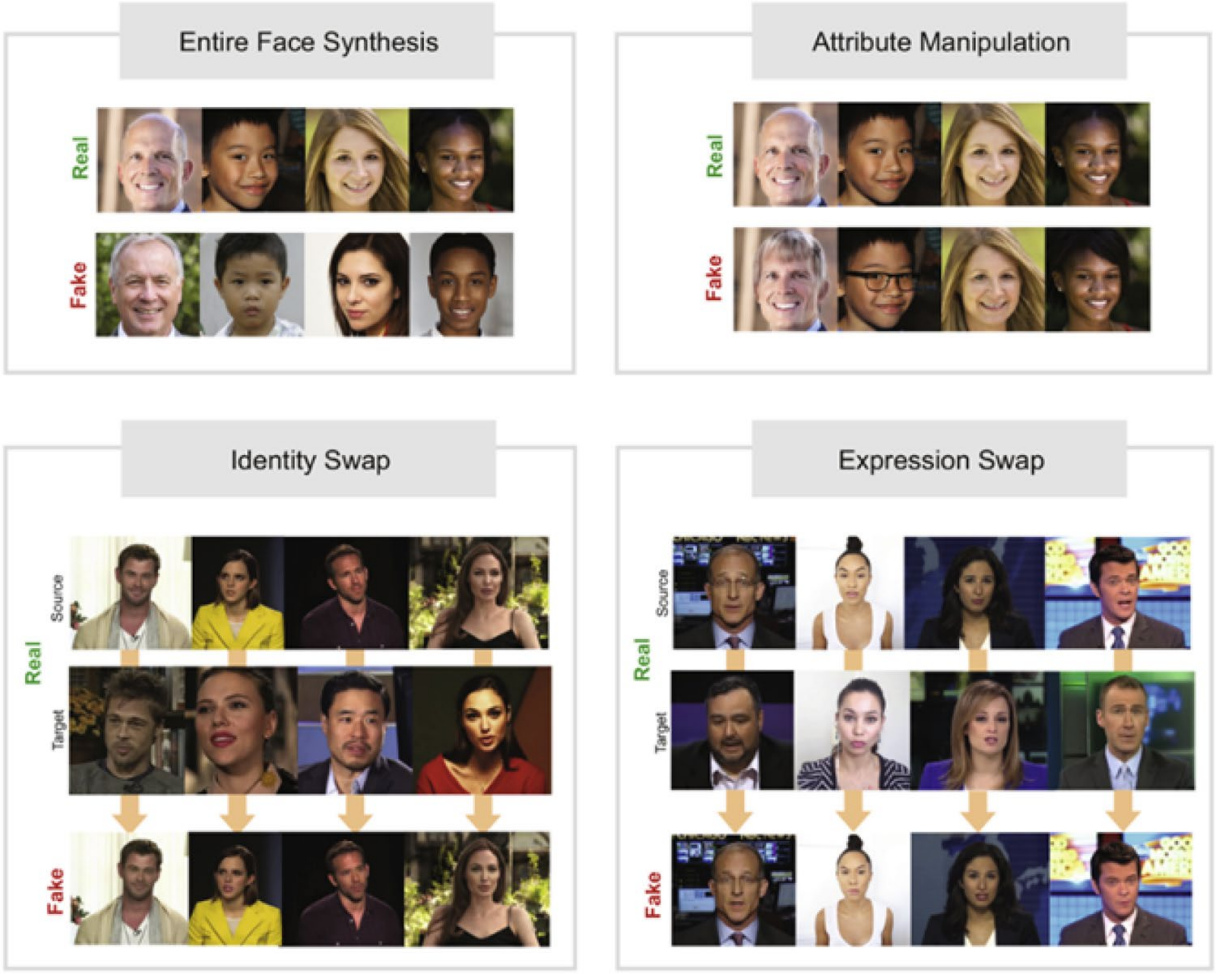
Real and manipulated image examples for each category of facial manipulation techniques^14^.

Figure 1 shows examples of original and manipulated images for each category as shown in Table 1. Facial manipulation techniques are being actively studied in all categories. In addition, datasets created using various facial image manipulation techniques such as FaceForensics++^7^, Celeb-DF^40^, DFDC^41^, and DeeperForensics^8^ have also been actively accumulated. Among the four categories of facial image manipulation described in Table 1, we focus on the application of expression swap in practical real-time situations.

Expression swap, also known as face reenactment, modifies facial expressions in images or videos by replacing the expressions of other faces into the target face. It can be divided into learning-based and animating-based approaches. Face2Face^30^, NeuralTextures^31^ and ReenactGAN^42^ are included in the learning-based approach. In Face2Face^30^, the authors proposed a real-time expression transfer method based on computer graphics for source and target videos. In NeuralTextures^31^, the authors combined the traditional graphics-based methodology with learnable components to express source and target videos as 3D representation and perform rendering-based facial reenactment. Both methods perform face reconstruction with 3D representation and learning of the model for a specific object. ReenactGAN^42^ transferred facial movements from one monocular image of multiple individuals to a specific target person. However, these methods also have the limitation of showing person-dependent performance.

On the other hand, the animating-based swap can be performed by extracting the changes in the facial expression in the target video and animating the facial expression of the source image using them. In FReeNet^43^, the authors proposed a multi-identity face reenactment framework to transfer facial expressions from an arbitrary source face to a target face. Similarly, Kong et al.^33^ introduced CFReenet, a controllable multi-identity face reenactment that uses a generation model after decomposing facial movements into poses and expressions. However, these models are limited to images and cannot be applied to videos. In the first order model^34^, the authors proposed a motion extraction-based model for face animation. It generates a new video by applying the motions extracted from a video to an appearance in the original source image. It does not require prior knowledge about the objects to be animated and training for the target objects. GANimation^36^ is a GAN-based image animation model that separates human expressions into action units, which are continuous movements of facial muscles. It maps a sequence of action units in a static image for representing the facial expressions and produces changes in adjacent areas through different combinations of action units. It showed high performance even with changes in background and lighting due to the use of attention mechanisms.

A previous study^37^ attempted to make the real-time transfer of facial expressions from a source video actor to a target video actor. The method used a predefined parametric model to represent facial expressions. Although it had high-resolution facial reenactment capability, it required two physical cameras with an RGB-D sensor to capture the facial expressions. In our study, we evaluate the feasibility of two representative expression swap models in an online real-time setting: the first order model^34^ and GANimation model^36^. Both models only require a source image and a target video as inputs and generate a manipulated video that animates the source facial expression based on the target video’s facial expression changes. This makes it easy to apply both models in real-time online environments.

## Evaluation framework for facial image manipulation

### Overall framework

**Figure 2:**
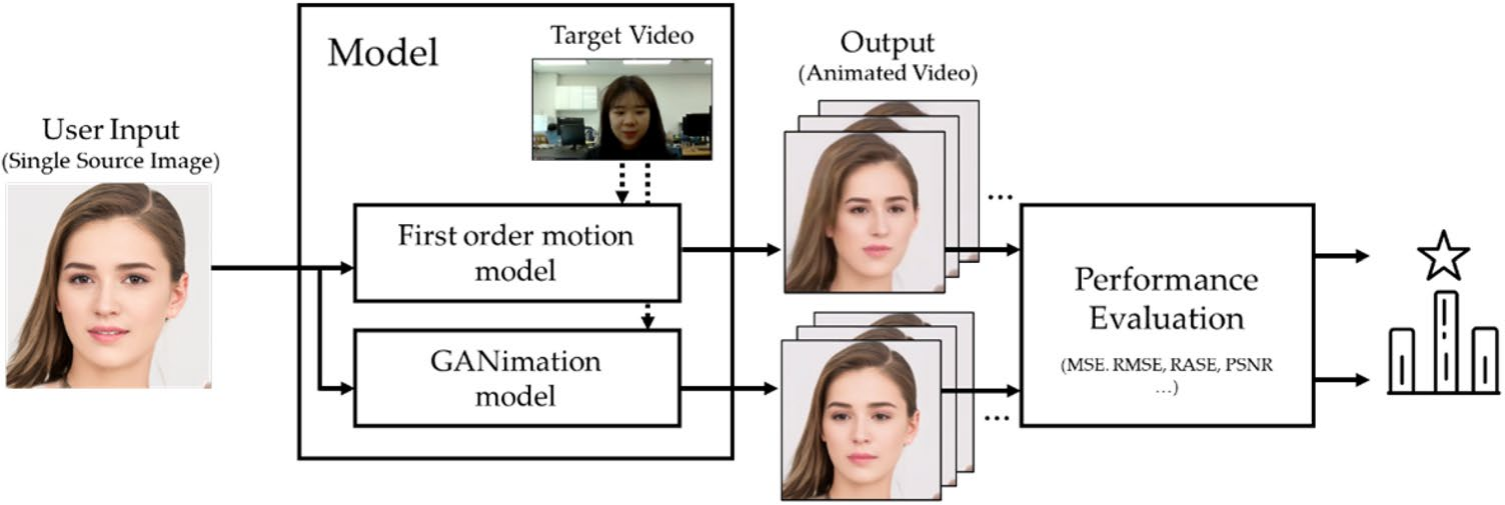
Framework for performance evaluation of animating-based expression swap.

Figure 2 shows the overall framework proposed in this paper for facial image manipulation, targeting real-time online class situations. Importantly, a single input image is fed into the model, considering the practical usage in online video conference applications. Then, the output is a video in which the expression changes according to the expression movement extracted from the target video. Finally, we measure the performance of the generated video by the frame quantitatively and qualitatively for the used models. In this study, we consider the two models for animating-based real-time face manipulation: (1) The first order model^34,35^ and (2) GANimation^36^.

### Model architecture

#### First order model^34,35^

**Figure 3:**
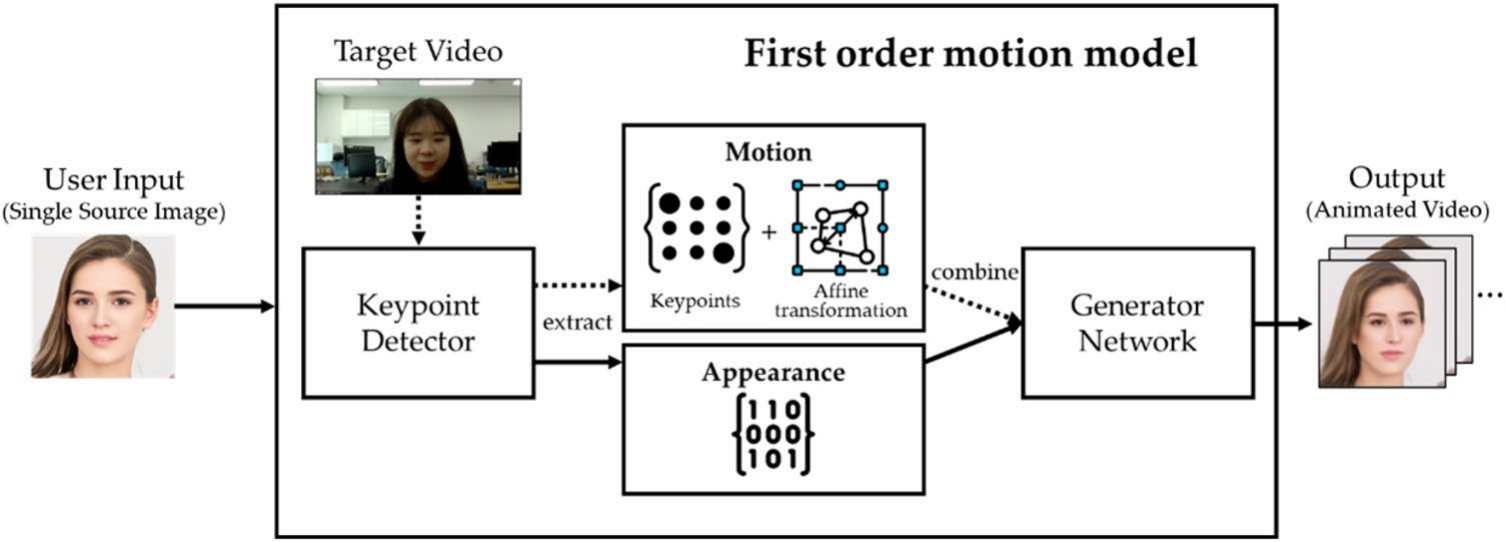
The first order model^34^ in our evaluation framework.

Figure 3 shows the architecture of the first order model tailored to our evaluation framework. It takes a source image and decouples appearance and motion information using a self-supervised formulation. They use a generator that models occlusions of motions in the target video and combines the appearance extracted from the source image and the motion derived from the target video. Through this process, a video animated from the source image’s face using the motion of the target video is generated.

#### GANimation^36^

**Figure 4:**
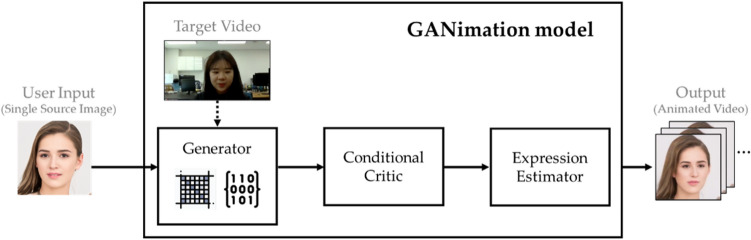
GANimation^36^ in our evaluation framework.

Figure 4 shows the architecture of GANimation. Hence, GANimation consists of three modules. (1) The generator module takes the original image and the target expression as inputs and then generates the manipulated image through regression operations using color and attention masks. (2) The critic module evaluates how realistic the generated image is. (3) The expression estimator module penalizes the residual between $y_g$, which are the real activated action units, and the calculated ${\hat{y}}_g$. Through this process, a manipulated video is generated so that the expression of the original image follows the target expression. In this study, we use a pre-trained model using CelebA dataset^44^, which uses the preprocessed dataset cutting only the face area. The limitation is that the performance of this approach is only acceptable when the portion of the face is similar to that of the faces in the pre-trained model even if it does not require a ton of images for the source or target. This is also confirmed in the experimental results of each scenario in “Scenarios for online class environments” section of this paper.

Both first order model and GANimation are animating-based facial image manipulation models that take one source image and one target video as the input. Then, they generate a video that animates the facial expression of the original source image according to the change of facial expression in the given target video, unlike other approaches that typically require thousands of images for the target and source. Therefore, we note that they are appropriate to be applied for real-time environments such as online classes.

### Scenarios for online class environments

**Figure 5:**
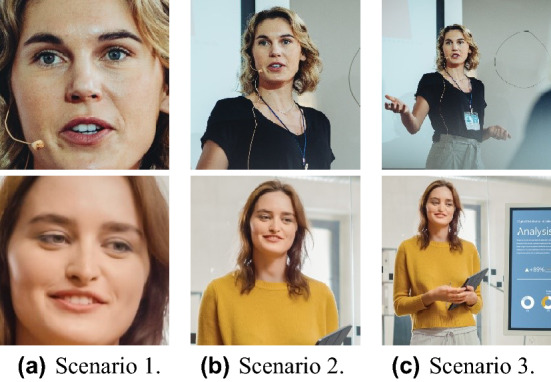
Sample images by the scenario.

In this study, we define the realistic situations in online classes based on video conferencing applications such as Zoom or Microsoft Teams as the following three scenarios: (1) attendance checks or listening to lectures, (2) presentations, and (3) examinations. By defining these different scenarios, we can differentially evaluate the performance of the models for image animating-based facial image manipulation. For each scenario, when one source image and target video are fed into the model, we can generate the video that animates the source image according to the expression extracted from the target video. Figure 5 shows the sample images by the scenario. We collected these facial images from shutterstock (https://www.shutterstock.com/), a free, copyright-free image website.

#### Scenario 1: attendance checks

The face is clearly visible while staring at the screen, and the proportion of face area occupies more than 50% of the entire space in the image, and the features are clearly visible.

#### Scenario 2: examinations

The upper body of the posture during the examination is visible along with the surrounding environment, and the proportion of face area occupies about 10% of the entire space in the image.

#### Scenario 3: presentations

The upper body or the whole body is visible and the proportion of the face occupies about 3% of the entire space in the image.

## Performance evaluation

### Experimental environments and methods

**Figure 6:**
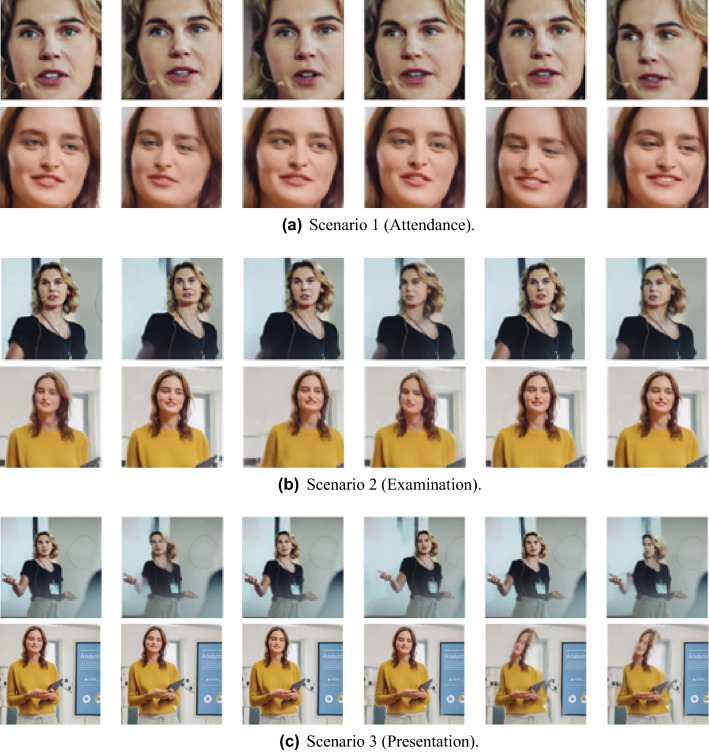
Sample frames of generated videos using the first order model.

**Figure 7:**
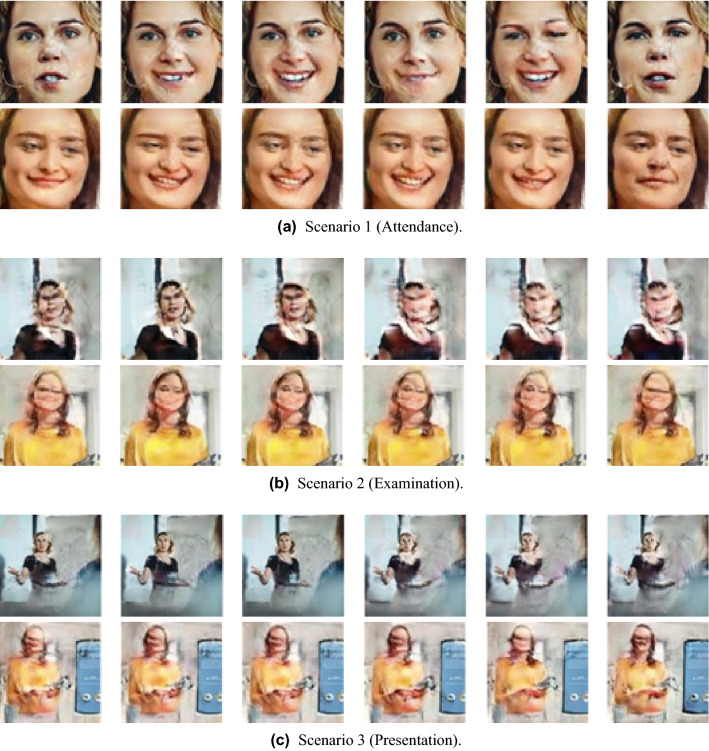
Sample frames of generated videos using the GANimation.

**Figure 8:**
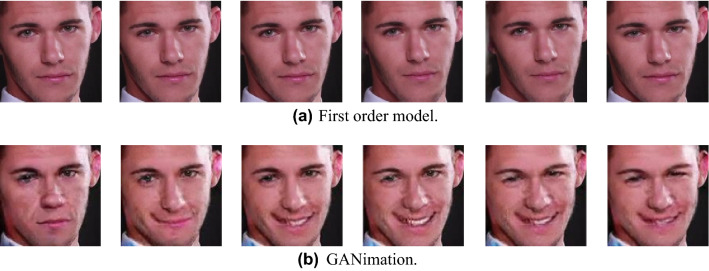
Differences between the two models when the facial expression changes.

To evaluate the quality of the facial image manipulated result, we selected three input source images and target videos for each defined scenario and performed an image quality assessment^45^ on videos generated using the animating-based face manipulation models. Image quality assessment (IQA) is a technique used to measure the quality of an image. In Deepfake detection, it is commonly used to evaluate the quality of manipulated image^45–47^. Nevertheless, IQA is not sufficient for the performance evaluation of the facial image manipulation results because it cannot catch the naturality in the target videos. Cognitive judgments by humans are required to complement its limitation. Therefore, we show the qualitative evaluation of the proposed framework in “Qualitative evaluation” section and discuss the IQA results observing actual examples in “Quantitative evaluation” section. We extract a total of 240 image frames, 80 frames from each target video, and evaluate the performance for frame-by-frame comparison. For the performance evaluation, we used an AWS EC2 p2.8xlarge instance equipped with Nvidia Tesla K80 with 12GB memory. We measure the performance by applying various evaluation indicators commonly used for IQA and distortion analysis to confirm the quantitative evaluation between manipulated and original images. The evaluation measures we used are shown in Table 2. We used the non-copyright image data of Shutterstock^48^ for the other input images in each scenario (Figs. 5,6, 7, and 8).

**Table 2:** Image quality assessment (IQA) metrics for evaluating performance.

#	Acronym	Name	Description
1	MSE	Mean squared error	It is a measure of the quality of the image estimate. The larger the value, the greater the degree of distortion of the image.
2	RMSE	Root mean squared error	It is used to evaluate the average performance of image fusion methods for each spectral band.
3	RASE	Relative average spectral error	It is used to evaluate the average performance of image fusion methods for each spectral band.
4	PSNR	Peak signal-to-noise ratio	It is used to measure the quality of reconstruction of lossy compression codecs. Higher PSNR means that the reconstruction is of higher quality.
5	UQI	Universal quality image index	It is designed by modeling any image distortion as a combination of three factors: loss of correlation, luminance distortion, and contrast distortion.
6	VIF	Visual information fidelity	It is based on natural scene statistics and the notion of image information extracted by the human visual system.
7	ERGAS	Relative dimensionless global error	It is used to estimate the overall spectral quality of fused images. It exhibits a strong tendency to decrease as the quality increases.

### Experimental results

#### Qualitative evaluation

Figures 6 and 7 show sample images of the video frames for each scenario generated using animating-based face manipulation models, respectively. Overall, it can be seen that the quality of the result after going through the model has decreased compared to the original images shown in Fig. 5. In the case of Scenario 1, both models naturally changed facial expressions, and the animation was well performed. Relatively, in GANimation, some unnatural image quality distortion is observed. In the case of Scenario 2, the first order model produces more natural animation of the object’s movements, such as turning its head, but no detailed changes in the movement of eyes and mouth, and some image distortion is observed. GANimation did not perform natural animation and showed a damaged result, which is difficult to recognize the face. In the case of Scenario 3, both models show unnatural changes in the movement of objects, and in particular, the quality degradation of GANimation becomes more severe than in Scenario 2.

#### Quantitative evaluation

Table 3 shows the quantitative performance evaluation results of manipulated videos for each scenario using two models. We evaluated the performance and calculated the average for each evaluation measure for 80 image frames extracted from each target video. As a result, the first order model shows better performance in Scenario 1 than GANimation as confirmed in quantitative evaluation. Among the evaluation measures, UQI is known to perform better in IQA than simple statistical error-based indicators such as MSE and PSNR. UQI for Scenario 1 of the first order model is 0.96, which indicates acceptable results when compared to the results reported in the previous study^49^. As such, Scenario 1 shows high performance, but when the proportion of the face becomes small, such as Scenarios 2 and 3, the performance greatly degrades, such as doubling in ERGAS.

GANimation also shows relatively acceptable performance in Scenario 1. However, its performance becomes severely degraded in Scenarios 2 and 3. In particular, MSE and ERGAS are significantly degraded, even 2 7 times worse than the first order model in Scenario 1, as confirmed in the qualitative evaluation results. We confirm that GANimation can be applied only in the situation where the face occupies a sufficiently large portion of the original image such as in Scenario 1. This stems from the fact that the used pre-trained model is trained using a dataset with images that have a similar portion of the face to Scenario 1. This indicates the limitation of GANimation: it depends on the pre-trained model. In other words, we need to accumulate enough datasets for each target scenario and build multiple pre-trained models for the target scenarios to achieve a high performance in GANimation, which is not required in the first order model.

**Table 3:** Performance evaluation of the expression swap models by the scenario on IQA metrics.

	Scenario	MSE	RMSE	RASE	PSNR	UQI	VIF	ERGAS
First order model	#1	**1202.27**	**32.16**	2342.35	**20.60**	**0.96**	**0.27**	**8715.36**
	#2	1613.37	39.67	NaN	16.26	0.85	0.16	21242.53
	#3	1492.89	37.79	NaN	16.26	0.85	0.15	39648.31
GANimation	#1	1916.28	40.44	**1243.96**	16.87	0.87	0.13	16408.81
	#2	3059.34	50.90	NaN	15.26	0.82	0.13	48698.43
	#3	2281.51	43.85	NaN	16.48	0.86	0.20	66222.71

Significance values are in bold.

According to the previous evaluation results, the first order model generally outperforms GANimation. Nevertheless, we investigate the case where GANimation outperforms the first order model. To confirm this, we focus on only the case where facial expressions are changed. Figure 8a and b show the manipulated results of the first order model and GANimation, respectively, when the target facial expressions are changed. As shown in Fig. 8a, in the first order model, we indicate that the direction or the size of the eyes change slightly, but no dramatic changes are observed in facial expressions such as smiling faces. On the other hand, as shown in Fig. 8b, in GANimation, we observe that the shape of the eyes and mouth changes dramatically, i.e., changing from an expressionless face to a smiling face. Therefore, we conclude that the advantage of GANimation is revealed when detailed facial expressions are changed in various ways while the first order model mainly focuses on smoothly converting overall motions and flows such as gaze direction and head position. As a result, even though the quantitative evaluation result of GANimation is lower than that of the first order model, in terms of facial expression change, the actual expression changes are better maintained in continuous frames.

## Demonstration

**Figure 9:**
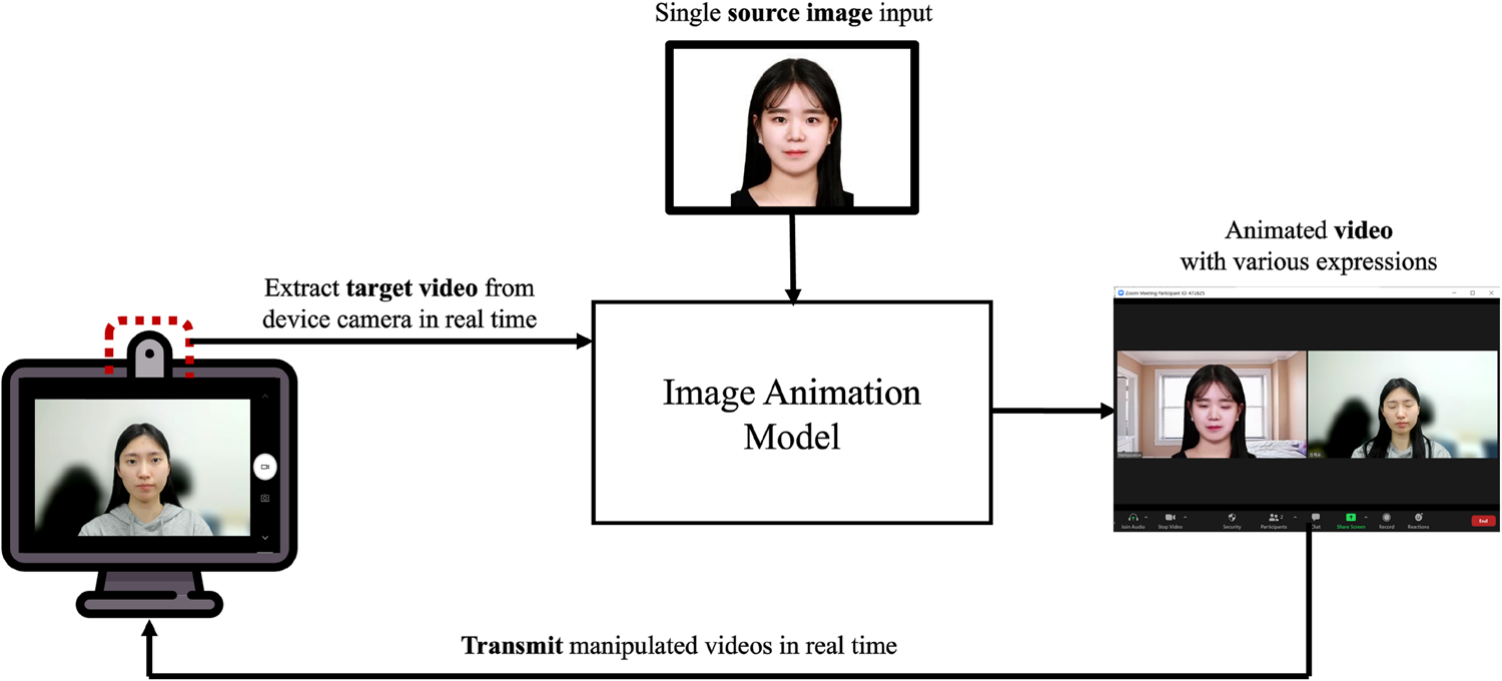
An architecture for demonstration in Zoom.

Figure 9 illustrates an architecture for the demonstration of applying the expression swap model into the Zoom platform to verify its real-time usability. Here, we used the first order model, which shows overall better performance than GANimation. We note that image manipulation is performed in real-time for a single source image and a target video recorded by the device’s camera. The animated video generated by the model is actually transmitted to the Zoom channel in real-time. In this way, the user can actually broadcast a virtual video using a manipulated image instead of his or her face taken by the device camera. We used the author’s own image for the target video and the source image input.

**Figure 10:**
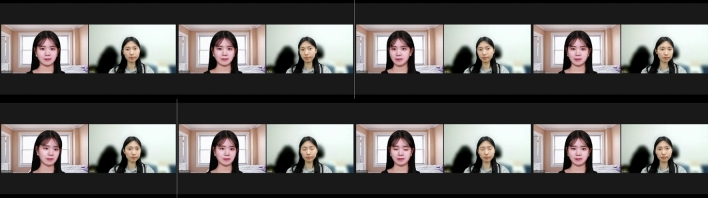
Demonstrated videos in Zoom.

Figure 10 is the actual demonstrated video in the Zoom environment. The right screen shows actual frames taken by the device camera, and the left screen shows the manipulated frames by the first order model in real-time. There is a delay between the actual video and the manipulated video, which can be seen from the flicker in the left screen of the sixth frame in Fig. 10 appearing on the right screen of the seventh frame. The time interval between frames is only about 0.1 seconds, which indicates a quite acceptable application for real-time service.

We demonstrate the extensive application of expression swap to online meeting platforms, including Zoom, Google Meet, and Microsoft Teams. To directly compare the results between a pair of platforms, we simultaneously run two platforms at a time. The model inputs a target image, and the manipulated video that follows facial expression changes captured by the device camera is broadcasted in real-time through the virtual camera.

**Figure 11:**
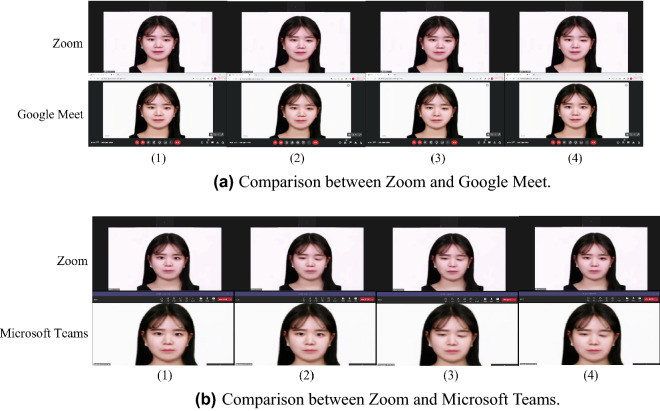
Comparison of expression swapping demonstration on Zoom, Google Meet, and Microsoft Teams.

Figure 11 compares the results between Zoom and Google Meet and between Zoom and Microsoft Teams using the first order model, which has a processing delay of 0.3 seconds. GANimation, with a processing time of 0.5 seconds, can also be applied in a similar way, but the results are not shown. Each frame was captured at 0.1-second intervals. In the comparison between Zoom and Google Meet, the flicker is observed in the fourth and third frames in Zoom and Google Meet, respectively; in the comparison between Zoom and Microsoft Teams, it is observed in the second and third frames, respectively.

## Conclusions

In this study, we proposed an evaluation framework of the expression swap models targeting real-time online class environments. Considering the application in the online class environments, the framework receives a single source image and a target video and generates the video that manipulates a target video where the face is replaced with the face in the source image. To this end, we selected two representative expression swap models: (1) first order model and (2) GANimation. We implemented these models in the framework and evaluated their performance for the defined scenarios. As an evaluation result, both models showed acceptable results in Scenario 1, where the face occupies a large portion of the screen. However, we observed that their performances are significantly degraded in Scenarios 2 and 3, where the face occupies less portion of the screen. In addition, it can be seen that the first order model causes relatively less loss of image quality than GANimation, so that more natural conversion is achieved. In contrast, GANimation model has the advantage of representing detailed facial expression changes compared to the first order model. Finally, we actually applied the expression swap model to the real online class environments using various online meeting platforms such as Zoom, Google Meet, and Microsoft Teams and showed its feasibility for real-time online classes. In particular, the first order model and GANimation model showed only 0.3 and 0.5 s of processing delay, respectively.

We have observed that animating-based facial image manipulation still has limitations in the application, in particular, when the proportion of the face in the image is small, such as in examinations or presentations. Due to the process of animating each frame after capturing the frame in real-time online video, the motions between frames are not naturally connected and the output video tends to be choppy. In addition, the first order model shows high performance in extracting the motion of an object, but has difficulties in extracting detailed facial expressions such as laughter and grimacing. As a further study, we plan to combine the distinguishing properties of other categories to the expression swap model, which has advantages in real-time application.

## Data Availability

All data from study can be access at https://drive.google.com/drive/folders/1SIdPg_ttDzDO3u0SXT1h834j3jyXY0bU. The uploaded video includes one of author’s face and it is permitted to be uploaded for open access publication.
